# On the Treatment of Chronic Inflammation of the Bladder by Injections of Nitrate of Silver

**Published:** 1849-07

**Authors:** R. L. McDonnell


*On the Treatment of Chronic Inflammation of the Bladder by Injections of Nitrate of Silver.*—By R. L. McDonnell, Esq., M. D.

In a paper, published in the Third Volume of this Journal, I drew the attention of surgeons, to the great utility of *Injections of nitrate of silver into the bladder, in chronic inflammation of that organ;* and, in support of my views, I adduced some remarkable instances of their successful employment, which had occurred both in my private and hospital practice. It is with the hope of placing this method of treating a disease, hitherto considered incurable, which one of the most eminent surgeons in the world—Sir Benjamin Brodie—considers the "opprobium of Surgery," and says, "there is no disease for which an improved method of treatment is more wanted," in that position in surgery, which, I feel convinced, it deserves to occupy, that I have laid the following cases before the profession.

Since my first paper was published, I have cured a great number of persons affected with this disease, but I have selected the following cases from amongst them, because, in them, the cure was *solely effected by the injections*—whereas, in some of the others, general treatment was likewise employed; and, in some, the affection of the bladder, was complicated with organic change of the prostate gland, with strictures of the utethra, and, in one instance, with urinary fistula and strictures—complications requiring special treatment, and which, some might suppose, assisted in relieving the affection of the bladder; although I am quite satisfied, the cures of the vesical inflammation, were due to the injections alone. I have also omitted some mild cases of the disease, because, as stated in my former paper, they might have been cured by remedies, generally known to surgeons, and, therefore, are not so valuable, as evidence in favor of the method by injections. But, my principal reason for selecting the following examples is, that we have in them, unquestionable testimony of the utility of the practice I advocate—for, in all, general treatment and the usual remedies *had been carefully and perseveringly employed, without success; and in one, the age of the patient, and the duration of the disease, were most unfavorable for testing the merits of the treatment, yet in all, the cure was complete and permanent.*

[Here follows a detail of the treatment referred to, but we have not thought it necessary to copy it.]

The following is Dr. M.'s directions for injecting the bladder.

The patient being placed either in the erect position or on a sofa, a gum-elastic catheter, about the size of No. 9 or 10 (Wiess,) is introduced, and water at the temperature of 98° Fahrenheit, is injected through this into the bladder, by means of a caoutchouc bag, or, what I prefer, a syringe, with a "three-way valve," by which the fluid can be drawn back from the cavity if necessary.—

After the bladder has been completely cleansed of any fetid urine and mucus which may be contained in it, the solution of the caustic, being heated to the same degree, is to be introduced in a similar manner, and allowed to remain there for about one minute, care being taken, by compressing the urethra, to prevent its being forcibly ejected by the violent straining that is certain to be induced. The quantity of water or solution should never exceed four ounces, for though the bladder in its healthy state is capable of containing nearly a pint and a half of urine, without being over-distended, yet as the quantity it is capable of retaining in severe chronic inflammation seldom exceeds a few tablespoonsful, the bladder accommodates itself to its diminished contents, and gradually becomes smaller, and consequently a large injection would act injuriously in two ways—by over-distending the organ or by passing up into the ureters. In fact, we find it unnecessary to use a larger quantity of the solution than I have mentioned, for it requires some address to introduce even that amount without resorting to force. The patient is then ordered a warm bath, and should the urine become bloody or mixed with shreddy concretions, he should use frequent fomentations and anodynes. But these symptoms seldom last for more than a few hours, and our patient should always be informed that such consequences are likely to be the immediate effects of the operation.

The strength of the injection has seldom to be increased beyond five grains to the ounce, although in one instance, that of an old gentleman, aged seventy-two, I had to increase the strength *gradually* to ten grains to the ounce before a satisfactory effect was produced. It is, however, always better to commence with a weak solution, which may be made stronger, according to the circumstances of each case, and the judgment of the practitioner. Some of my patients have hesitated about undergoing treatment by injections, in consequence of their advanced age, but though the disease is not in such cases so easily cured, as in the young subject, it is still, in the great majority of instances remediable by the same means as was proved by the great relief obtained by a patient aged *seventy-six*, who was under my care in the Montreal General Hospital, within the last month, into whose bladder I injected, on two occasions, a solution of nitrate of silver, two grains to the ounce. He left the Hospital of his own accord, May 23, quite free from his former complaint.

The Surgeon should, in fact, show his patient that all general treatment and local remedies having failed, he has only two alternatives to choose between—a life of misery and suffering, a burthen to himself, and incapable for the enjoyment of society, or the performance of business—and submission to a plan of treatment, which has been eminently successful in cases equally protracted and aggravated as his own, and in patients equally old and infirm, and who like him had spent time and money, and exhausted their patience, in ineffectual efforts to get rid of a disease so formidable, so excruciating, and so disgusting to themselves and others, as Chronic Inflammation of the Bladder.—*British Am. Med. Jour. Phys. Sci.*

